# The Book World of Medicine and Science

**Published:** 1906-02-10

**Authors:** 


### THE BOOK WORLD OF MEDICINE AND SCIENCE.

The Water Supply of Villages and Small Towns.—By H. C. H. Shenton, M.S.E. Reprinted from the "Local Government Journal." (Price 6d.)

In this little book, or, rather, pamphlet, Mr. Shenton supplies "practical notes in a handy and portable form" concerning the important subject of which he treats; and he enumerates, rather than describes, the chief methods and appliances which may be used for the establishment of a water supply. The book is nowhere sufficiently comprehensive to enable owners or authorities to dispense with professional advice; but it is nevertheless full of condensed information, and would often afford valuable guidance as to the directions in which water should be sought, and as to the methods by which it may be rendered available for domestic requirements.

